# Miscellaneous Notices

**Published:** 1850-01-01

**Authors:** 


### Miscellaneous Notices.

*Physiognomy of Diseases.* By GEORGE CORFE, M.D.

IT was our intention to have made Dr. Corfe's valuable contribution to medical literature the basis of a separate article in the body of the journal. Press of matter alone has prevented us from carrying this intention into execution. We must defer the publication of the article referred to, until the next number of the journal. We cannot, however, go to press without directing public and professional attention to Dr. Corfe's work. It will repay perusal.

---

*On Tic Douloureux, and other Painful Affections of the Nerves, with Suggestions for their Treatment by means of the Aneuralgicon.* By C. TOOGOOD DOWNING, M.D., &c.

DR. DOWNING has, for some years, been directing his attention to the pathology and treatment of tic douloureux, and the results of his labours are embodied in the work before us. It does not constitute a bulky tome. Its typographical pretensions are of a humble character; but, nevertheless, it has its value. A great book is said to be a great evil. The author's description of the symptoms and characteristics of tic douloureux is graphic, and true to nature. On the subject of treatment, it is gratifying to hear an experienced physician declare, in answer to the question, Can tic douloureux be cured?

"I believe it can. I will not pretend to assert that cases may not occasionally arise which will defy all human skill; yet, in my opinion, the greater number might be cured—certainly their sufferings might be considerably alleviated."

The *aneuralgicon*, which Dr. Downing has invented for the treatment of this, and other painful affections of the nerves, is a fumigating apparatus, in which dried herbs are burnt, and the heated vapour directed to any part of the body. The author says:—

"The materials used in the aneuralgicon are chiefly the leaves, slender stalks, and seeds of plants. After carefully selecting the herbs, to ascertain their genuineness and purity, they are thoroughly dried by a gentle heat

Each leaf, if it be a large one, is then taken separately and rubbed between the hands, so as to break up the parenchyma into small fragments, from which all stalks and woody fibre should be excluded. Some roughly powdered cascarilla bark may then be added with advantage.

"The plants I have chiefly employed have been those of the belladonna, henbane, cannabis indica or Indian hemp, tobacco, aconite, stramonium, hemlock, savine, digitalis, and a few others. The seeds of henbane, colchicum, and cannabis, have also been added under certain circumstances.

"The chief medicinal effects I have noticed in the use of this instrument are those of a sedative character. But the remedial influence of the aneuralgicon is not alone confined to the use of certain herbs. A considerable power is attributable to the warmth or intense heat generated. When the vegetable matter is ignited, and a current of air is made to pass through the burning mass, a small or great degree of heat can be produced at pleasure. Thus, when the hand is gently pressed upon the bellows, a mild, warm stream of vapour is poured forth, which may act as a douche to tender parts. But by strongly and rapidly compressing the same receptacle, the fire within the cylinder is urged, like that of a smith's forge, and the blast is intensely hot and burning. In this way any degree of rubefaction may be effected on a large or small surface, and by gradually augmenting the temperature, no bad substitute for the moxa is obtained. Thus we have in this aneurodyne apparatus the effects of heat and of medicated vapour; and each of these may be obtained singly, or combined together in regulated proportion.

"In allaying the pain dependent on excessive irritability and excitability of particular nervous fibrils, the aneuralgicon will be found a most valuable auxiliary, sometimes a direct therapeutic agent. The effects of both gentle heat and sedative vapour combine to produce the cure."

The concluding part of Dr. Downing's essay consists of the record of cases, for the cure or alleviation of which the instrument has been successfully employed. We are much pleased with Dr. Downing's work. It gratifies us to see the absence of everything savouring of empiricism. We strongly recommend our readers to read the work, and to try the mode of treatment which the author recommends.

---

*First Principles of Medicine.* By A. Billing, M.D., F.R.S., &c. Fifth Edition. 1849.

We are rejoiced to see Dr. Billing's valuable work fully appreciated. It has now reached a fifth edition, and comes before us stamped with the commendation of the highest authorities of this and foreign countries. We can call to mind the delight and profit with which we read the early edition of this treatise, and we hail the present volume with the same fervour that we should greet an old and much-valued companion of our early days. The present edition has been carefully revised by the author. It contains much additional valuable matter, and the alteration in the arrangement also calls for our warm eulogy. There are parts of the work which we should, if we had space, like to extract. We must defer this pleasure to another occasion.

---

*Lectures on Electricity and Galvanism, &c., delivered at the Royal College of Physicians.* By Golding Bird, M.D., F.R.S. London: 1849.

The deservedly high position of the author of this work is sufficient to guarantee the careful attention of the profession to anything that proceeds from his pen. The author considers, in the early chapters of his work, the history of his subject, the origin of animal electricity, the sources of animal heat, the construction of the electric machine, the action of electricity on contractile tissues, and the application of this agent in the treatment of various diseases. This is one of the most scientific and learned works we have ever read on the subject of electricity and galvanism, in all their important theoretical and practical bearings. We particularly direct the attention of our readers to those portions of the volume having a special reference to the nervous system in health and disease. Dr. Bird entertains a high opinion of the application of this agent in the cure of certain local nervous affections.

---

